# Production of β-Glucans by *Pleurotus ostreatus*: Cultivation and Genetic Background

**DOI:** 10.3390/ijms26199703

**Published:** 2025-10-05

**Authors:** Jakub Drężek, Justyna Możejko-Ciesielska

**Affiliations:** 1 Department of Microbiology and Mycology, Faculty of Biology and Biotechnology, University of Warmia and Mazury in Olsztyn, 10-719 Olsztyn, Poland; kubadrozek@o2.pl; 2 Chemprof Doradztwo Chemiczne s.c. Katarzyna Łuczyńska i Michał Łuczyński, 11-041 Olsztyn, Poland

**Keywords:** bioactive compound, β-glucan, health benefits, *Pleurotus ostreatus*, proteomic, transcriptomic

## Abstract

*Pleurotus ostreatus* is one of the most frequently cultivated mushroom species. It has attracted considerable attention not only because of its short cultivation time, but also because of the wide range of substrates on which it can be cultivated, such as lignocellulosic materials, synthetic polymers and wastewater. The popularity of the oyster mushroom stems not only from its rapid growth and high adaptability, but also from its functional ingredients, which include laccase, proteoglycan and β-glucan. As understanding the molecular biology of *Pleurotus ostreatus* is crucial for evaluating its commercial and scientific applications, modern molecular tools have been used to search for the genes and proteins involved in the development of this mushroom and production of valuable metabolites. The rapid development of artificial intelligence may make it possible to automate and optimize the entire cultivation process of *Pleurotus ostreatus*. This report summarizes the cultivation of *Pleurotus ostreatus* using waste raw materials, the nutritional and medicinal value for applications, transcriptomic and proteomic analyses and the use of artificial intelligence systems. In addition, future perspectives are discussed to make the cultivation of *Pleurotus ostreatus* environmentally friendly and to ensure an increase in its productivity and quality.

## 1. Introduction

**Figure 1:**
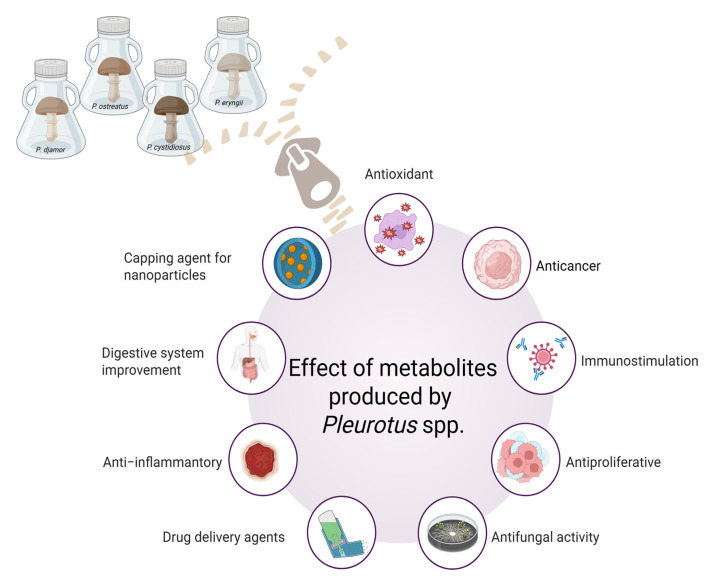
Overview of bioactive metabolites properties produced by the representatives of *Pleurotus* spp., e.g., *P. ostreatus*, *P. eryngii*, *P. cystidia’s* and *P. djamor* (prepared with Biorender.com).

Biologically active components are gaining attention due to their pharmaceutical properties for medical applications. In recent years, particular attention has been paid to edible mushrooms, which are widely used in traditional and functional foods [1]. Worldwide mushroom production increased from 31.78 million tons in 2012 to 50.01 million tons in 2023 [2]. This phenomenon is reflected in the global market value of functional mushrooms, which is estimated at USD 12.34 billion [3]. The most commonly cultivated mushroom species today are the white button mushroom (*Agaricus bisporus*), the oyster mushroom (*Pleurotus* spp.) and the shiitake mushroom (*Lentinula edodes*) [4]. However, if all the necessary conditions are met, the *Pleurotus* grow the fastest and are the easiest to cultivate. They grow rapidly at temperatures between 10 °C and 30 °C and in a medium with a pH between 6 and 8 [5]. Moreover, *Pleurotus* spp. known as edible mushrooms wigastronomic, nutritional, and medicinal properties (Figure 1).

*P. ostreatus* is the most interesting *Pleurotus* sp. that can be grown on a variety of wastes [6]. The high enzymatic adaptability of *P. ostreatus* allows it to grow on different lignocellulosic substrates, which in combination with its rapid growth and high-yield potential, makes the cultivation of the oyster mushroom easy and cost-effective [7,8]. It is also known for its unique properties, especially in terms of phytochemical content and antioxidant properties [9]. The oyster mushroom was first described in 1774 by Nikolaus Joseph von Jacquin. Due to its macroscopical characteristics, *P. ostreatus* was classified in the genus *Agaricus*, but Kummer [10] transferred this species to the genus *Pleurotus*. The Latin name of oyster mushroom, *P. ostreatus*, refers to the shape of its fruiting body. The genus name (*P.*) means “side-ear” and refers to the stem, which grows sideways in relation to the cap. The species name (*ostreatus*) refers to the shape and texture of the oyster [11]. *P. ostreatus*, the largest edible mushroom, has attracted much attention in recent years and requires more in-depth research. Recently, Nakazawa et al. [12] discussed advances in the molecular genetics of *P. ostreatus* and demonstrated its status as a model fungus for the study of wood degradation, sexual development and cell wall structure. However, a comprehensive overview of the cultivation of *P. ostreatus* using waste raw materials, its nutritional and medicinal value for applications, transcriptomic and proteomic analyses and the use of artificial intelligence systems in the cultivation of *P. ostreatus* is lacking. Therefore, this review emphasizes the recent progress and challenges in the above-mentioned topics on *P. ostreatus*. Future perspectives are also discussed to make the *P. ostreatus* cultivation process environmentally friendly and ensure an increase in productivity and quality.

## 2. Cultivation of *Pleurotus ostreatus*

### 2.1. Effect of Culture Media on the Growth and Biomass

The first critical step for successful mushroom cultivation is to nurture the mushroom and to ensure their optimal growth before transferring them to the substrate. Meeting the nutrient requirements of the mycelium ensures rapid vegetative growth, which directly contributes to a higher final yield. A detailed study of the effects of different culture media on the growth of *P. ostreatus* on an in vitro scale enables better optimization of the entire cultivation process. This approach enables a more detailed understanding of the physiological responses of the fungus to different carbon and nitrogen sources, facilitating the development of more efficient cultivation strategies.

Pant et al. [13] investigated the effects of five different solid culture media on the growth of *P. ostreatus*, e.g., Potato Dextrose Agar (PDA), Oat Meal Agar (OMA), Malt Extract Agar (MEA), Corn Meal Agar (CMA) and Wheat Extract Agar (WEA). It was found that the PDA medium allowed the most expansive growth, resulting in a mycelium diameter of 8.38 cm on the 10th day of incubation. This was followed by MEA medium (7.72 cm mycelium diameter on day 10) and WEA (7.33 cm mycelium diameter on day 10). These results agree with those of Sardar et al. [14] who confirmed that PDA medium is the most suitable medium not only for *P. ostreatus*, but also for *P. sajor caju, P. sapidus, P. columbinus* and *P. eryngii*. In a study conducted by Fletcher et al. [15], the influence of PDA medium supplemented with Yeast Extract (PDYA), Sabouraud Dextrose Agar (SDA) and Iron Sulphide Agar (ISA) were tested. The results showed that the PDA medium was best suited for the growth of *P. ostreatus* and allowed the development of a mycelium with a diameter of 23.28 cm. Despite additional supplementation, growth on PDYA resulted in a smaller diameter mycelium (14.73 cm). Hoa and Wang [16] found that Yam Dextrose Agar (YDA) supported the growth of *P. ostreatus* better than PDA medium; however, the difference in growth on these two media was not significant.

The oyster mushroom can also be successfully cultivated in liquid media by submerging it. The mycelium obtained with this cultivation method can be used as a source for the extraction of valuable bioactive compounds. The fulfillment of nutrient requirements in liquid cultures is therefore directly related to the yield of these compounds [17]. Nidhi and Sud [18] investigated the influence of Potato Dextrose Broth (PDB), Potato Dextrose Yeast Broth (PDYB), Malt Extract Broth (MEB), Czapek’s Dox Broth (CDB), Potato Malt Broth (PMB), Corn Meal Broth (CMB), Ashby’s Mannitol Broth (AMB), Yeast Extract Broth (YEB), Yeast Malt Broth (YMB) and Potato Sucrose Broth (PSB). It was found that PDYB was the most optimal medium for liquid culture, followed by YMB. The lowest growth was observed on CMB. However, liquid media enriched with additional nutrient sources are more effective. Krupodorova et al. [19] demonstrated that although the growth of *P. ostreatus* can be maintained on Glucose-Peptone Yeast Broth, the addition of plant waste material can increase the yield almost threefold. This was achieved by adding amaranth flour and broken vermicelli, and the wheat germ cake also led to a significant increase in yield compared to the control group. Similar studies by Lee et al. [20] have shown that not every plant waste material can be successfully used as an alternative to a typical mycological liquid medium. When comparing Potato Dextrose Broth (PDB) with 15 different liquid media containing 0.3% soybean meal and sugar in varying concentrations, it was found that PDB medium was the most optimal for the growth of *P. ostreatus*.

The influence of culture media on growth rates and biomass can be strain-specific, requiring customized approaches for different *Pleurotus ostreatus* isolates [21]. In addition to supporting growth, the composition of the culture media also affects the production of extracellular enzymes such as laccase, which are important for the degradation of lignocellulose and substrate utilization during cultivation. Media with a high content of proteins and non-reducing sugars are associated with increased enzyme activity, but do not always correlate directly with maximum biomass production, suggesting a trade-off between growth and production of secondary metabolites [22]. Overall, media with balanced macro- and micronutrients, an appropriate carbon-to-nitrogen ratio and suitable physical properties promote optimal growth of *Pleurotus ostreatus* and biomass accumulation.

### 2.2. Effect of Culture Conditions on the Growth and Biomass

In addition to nutrients, other environmental conditions also have a significant influence on the development of *P. ostreatus* during incubation. Even though the general ranges of parameters such as pH of the medium, temperature of incubation, CO_2_ concentration and light intensity are known and based on observations from the natural habitat of the oyster mushroom, precise values are necessary to maximize yields.

In the in vitro culture of oyster mushrooms, the optimal temperature was determined by Hoa et al. [16]. *P. ostreatus* cultured in PDA medium and incubated at 6 different temperatures (16 °C, 20 °C, 24 °C, 28 °C, 32 °C and 36 °C) grew fastest at 28 °C, followed by 32 °C and 24 °C. Hu et al. [23] investigated the influence of eight ambient temperatures (15 °C, 18 °C, 20 °C, 22 °C, 25 °C, 28 °C, 30 °C and 32 °C) on the growth, development and yield of *P. ostreatus.* It was confirmed that the highest growth rate of oyster mushrooms is achieved at 28 °C. The shortest time of primordia formation was also observed at 25 °C and 28 °C. The highest activity of the enzymes that degrade lignin and cellulose was observed in the early stages of development at temperatures between 15 and 22 °C and gradually decreased with increasing temperature. Considering that enzyme activity is directly related to the yield of *P. ostreatus*, it was concluded that the optimum temperature for growing this fungus is 22 °C.

The pH of the culture medium has a significant influence on the biological processes of the oyster mushroom, as it directly affects enzymatic activity, nutrient uptake and overall development of the mycelium by influencing cell metabolism. Sardar et al. [14] investigated the effects of PDA media with different pH values (pH 4, 5, 6, 7, 8) on the growth of *P. ostreatus*. It was found that the most optimal pH was 6, followed by pH 7, while the least optimal pH was 4, followed by pH 8. In a study conducted by Pant et al. [13] using the same medium (PDA) at the same temperature (25 °C) and similar pH ranges (pH 5, 6, 7, 8, 9), pH 7 was found to be the most optimal value. The statistical differences in growth between pH 6 and 7 were not significant in both of the above-mentioned investigations and could be due to the different pH preferences of the strains. Cultivation of *P. ostreatus* can also be performed to obtain valuable bioactive compounds. One of the most common of these compounds is laccases. Díaz et al. [24] tested the influence of four different pH values (pH 3.5, 4.5, 6.5 and 8.5) of the liquid medium used for the submerged cultivation of oyster mushrooms. It was found that the highest laccase activity was observed in cultures with a pH of 4.5 and the lowest at a pH of 8.5. This discrepancy emphasizes the need to adjust the environmental parameters of the incubation to the desired results of the *P. ostreatus* culture.

Since the mushroom’s cells respire, adequate ventilation is necessary to avoid high carbon dioxide concentrations in the growing room. Studies by Lin et al. [25] have shown that a CO_2_ concentration of 1% leads to shorter and thinner stems, inhibition of the sexual differentiation process and down-regulation of elongation factors. The optimization of CO_2_ concentration in the fruiting chamber was carried out by Jang et al. [26]. Four CO_2_ concentrations were tested (0.03%, 0.1%, 0.3% and 0.5%). It was confirmed that the concentration of 0.3% was the most optimal, while the mushrooms showed signs of deformation at 0.5% and above.

Although light is not necessary for the vegetative growth of *P. ostreatus*, it is essential for the initiation and growth of fruiting bodies, so the right light conditions are crucial to obtain a high yield of good quality. Siwulski et al. [27] tested four different light levels (100, 300, 500 and 700 lx) during three lighting periods (6, 10 and 14 h of light per day). It was found that the highest yield was achieved with a light intensity of 500 and 700 lx during a photoperiod of 14 h per day. The lowest yield was achieved at 100 lx during the 6 h photoperiod. Light intensity and photoperiod are not the only lighting parameters that significantly influence the development of the fruiting bodies of oyster mushrooms. De Bonis et al. [28] investigated the effects of three different wavelengths (450 nm, 610 nm and their combinations) on the agronomic properties of *P. ostreatus*. It was found that the application of light with a wavelength of 610 nm promoted the growth of the mycelium, while 450 nm stimulated the diametrical growth of the fruiting bodies. In addition, the vitamin D2 concentration in the fruiting bodies was higher both at 450 nm and at combined wavelengths.

To maximize the growth and biomass of *Pleurotus ostreatus*, culture conditions must be precisely controlled and optimized by balancing temperature, humidity, pH and substrate properties. Understanding strain-specific tolerances and responses is important for adapting cultivation methods to local environments and production goals. This knowledge enables improvements in yield, quality and sustainability of oyster mushroom production.

### 2.3. Effect of Various Spawning Doses

Another important factor in the cultivation of oyster mushrooms is the choice of the correct spawning rate when inoculating the growth substrate. Adjusting the spawning rate can affect both the speed of mycelium development and the quality of the yields obtained. It is important to find a balance between spawning rates, as too much spawn on a single substrate increases production costs, while too little can hinder mycelium growth and thus reduce yield.

Using the highest possible dose of spawn seems to be the relatively safest and most optimal method of selecting the amount of spawn for inoculation. This is also confirmed by the studies of Dahmardeh [29]. When cultivating *P. ostreatus* in polythene bags with three different amounts of spawn (2.5%, 4% and 5% of the wet weight of the substrate), the highest yields were achieved with the highest amount of spawn. A similar result was achieved by Pal et al. [30] who achieved the highest yields, the highest biological efficiency and the shortest spawning times with the highest applied spawning rate (8%). Considering the performance of lower doses and the total cost of spawn, a spawning rate of 2 to 4% was found to be sufficient for growing oyster mushrooms. The use of lower amounts of spawn can also be beneficial from an economic point of view. In a study by Bhatti et al. [31], 10, 20, 30, 40, 50, 60, 70, 80, 90 and 100 g of spawn per kg of dry substrate were used. It was found that the shortest period between fertility spurts (6.44 days) was achieved with a spawn of 20 g, but the application of 100 g of spawn allowed the highest number of fruiting body clusters per bag (7.30). However, a spawn amount of 70 g per kg of dry substrate performed best, as it had the highest fresh weight (45.4%) and dry weight (4.63%) in this group. In addition, the group with 70 g of spawn had the highest number of fruiting bodies per bundle. These results are in contradiction with the results of Idowu et al. [32] who tested similar amounts of *P. ostreatus* spawn (3%, 5%, 7%, 9%, 11% and 13%). The shortest time for substrate colonization and primordia emergence (19.67 days and 32 days, respectively) was observed at a spawning rate of 13%. The highest weight of fruiting bodies per bag (65 g/bag) was also found at 13%. At a spawning rate of 11%, the differences in these parameters (i.e., time of colonization, appearance of primordia and weight of fruiting bodies per bag) were smaller, but no significant difference was found between 11% and 13% spawning rate. The high spawning rates of over 10% in this study could be due to the use of the top and bottom method of inoculation, as opposed to the topical spawning method most commonly used in other studies.

The spawning dose is a decisive parameter for the cultivation of *Pleurotus ostreatus*. A moderate increase in spawning dose accelerates colonization, reduces the risk of contamination and maximizes yield and biological efficiency up to an optimal level, beyond which diminishing returns and increased risks occur [29]. Careful calibration of the spawning rate supports efficient and profitable mushroom production.

## 3. Cultivation of *P. ostreatus* on Waste Substrate

The robust enzymatic activity of *P. ostreatus*, the low nutrient requirements and the relatively short spawning and fruiting phase allow it to be cultured on a variety of substrates, including waste materials. In addition, the cultivation of oyster mushrooms also enables the beneficial utilization of otherwise harmful materials. The reuse of spent mushroom substrates in new cultivation cycles reduces the amount of waste and promotes a circular economy. This not only addresses environmental concerns but also improves resource efficiency in mushroom production.

### 3.1. Food and Agricultural Waste

Continuous research on biotechnological applications of *P. ostreatus* has shown that the cultivation of this fungus on various types of waste materials is not only possible but can also support higher yields compared to conventional growth substrate [33]. The most commonly used waste materials for the cultivation of *P. ostreatus* are agro-industrial residues (Table 1). These include sawdust, cotton cake, coffee residues, barks and straws. Each of these low-value agricultural wastes can be successfully used for the cultivation of oyster mushrooms [34].

Akcay et al. [44] demonstrated that hazelnut branch cuttings, hazelnut husk, wheat straw, spent coffee grounds and rice husks can be used as substrates for the cultivation of *P. ostreatus*. The highest yield value and the highest biological efficiency were determined when cultivating with hazelnut branch pruning wastes (255.7 g/kg and 63.9%, respectively). In a study by Jafarpour et al. [48], the possibility of growing *P. ostreatus* on food waste such as carrot pulp, soya cake powder, rice bran, wheat bran and a combination of soya cake powder and rice bran was investigated. It was found that the combination of commonly used agricultural wastes in the form of rice bran and soya cake powder produced the highest biological efficiency (127.8%) and yield (638.8 g/kg), with the shortest spawning time (35.8 days) achieved with pure rice bran substrate. Fufa et al. [35] tested corncob, finger millet straw and bamboo waste as possible substrates for oyster mushrooms. The highest yield (253.07 g/0.5 kg) and the highest biological efficiency (50.2%) were found for cultures on finger millet straw. The shortest spawning period (28.71 days) was observed on corncob substrate.

The most efficient way to utilize certain food waste for the cultivation of *P. ostreatus* is to use it as a supplement to growth substrates. In a study by Ahmed et al. [42], the ratio of tea waste to sawdust and tea waste to rice straw (75%:25%, 50%:50%, 25%:75%) was investigated in the cultivation of oyster mushrooms. It was found that the use of 75% tea waste in the substrate composition stopped the development of the mycelium. When tea waste was added to rice straw, the yield and biological efficiency were lower in all other experimental variants than in the control group (i.e., 100% rice straw). However, in the case of sawdust, the 50% tea waste fraction showed the highest yield (189 g/kg) and the highest biological efficiency (79%) of all tested variants. Alsanad et al. [45] came to a similar conclusion when they tested different ratios of wheat straw and spent coffee grounds (100%:0%, 67%:33%, 33%:67% and 0%:100%). Similarly to the aforementioned studies, a high (i.e., 100%) proportion of food waste in the form of coffee grounds did not allow mycelium to develop and had to be discarded. The highest values for biological efficiency and yield were found for substrates with a proportion of 100% wheat straw and 67% wheat straw to 33% spent coffee grounds (105%, 910 g/bag and 105.1%, 814.6 g/bag, respectively), although the differences between the two were not significant.

The addition of food waste can not only improve the yield of commonly used substrates but also enable the utilization of waste materials from other sources. Ma et al. [49] investigated the possibility of combining used cellulose nappy cores and food waste (coffee waste, sugarcane bagasse, banana peels and eggshells) into an efficient substrate for the cultivation of *P. ostreatus*. There was no significant difference in spawning time (31 days) in the variant that contained the largest proportion of the diaper core in combination with food waste compared to the control experiment with commercial mushroom cultivation block (32 days). In addition, the highest biological yield (84 g/600 g) was observed in the cultivation with the used diaper, coffee waste, sugarcane bagasse, banana skin, and eggshell at the ratio of 5:2:1:1:1. The results confirmed the suitability of nappy cores and food waste as a potential growth substrate for oyster mushrooms.

Mushroom cultivation itself generates waste in the form of spent mushroom substrate (SMS). Wang et al. [46] studied the use of different ratios of SMS of industrial *Hypsizigus marmoreus* in the *P. ostreatus* culture. The addition of 12% SMS was found to yield the highest total fresh weight (213 g/kg) and the highest biological efficiency (61.21%). Furthermore, the shortest mycelial colonization time was observed in a treatment with 88% SMS. Similar results in the cultivation of *P. ostreatus* were obtained by Dedousi et al. [50], who tested the application potential not only of SMS, but also of fresh mushroom compost (CM) and spent mushroom compost (SMC) of *Agaricus* mixed with *P.* and *Agaricus* waste. The shortest spawning time (29 days) was observed in the cultivation with the substrate consisting of 80% SMS and 20% mushroom waste (stipes/misshapen mushrooms) from the *P. ostreatus* culture. The authors showed that commercial SMS (GZ), either alone or in combination with mushroom waste supplemented with residues and by-products from the mushroom industry, are the most promising alternative substrates for the cultivation of *Pleurotus.* spp. to achieve high BE (up to 80%) in combination with a short total cultivation time.

Due to the robust enzymatic activity of *P. ostreatus*, it is possible to cultivate this fungus in highly contaminated environments. It has been proven that the oyster mushroom can be successfully used for the mycosanitation of pharmaceutical and industrial wastes, chlorinated pesticides and solid petroleum wastes. However, depending on the type of pollutant, it is important to evaluate the accumulation of the remediated waste in the fruiting bodies [51]. One of the most common pollutants affecting agricultural production are mycotoxins. The USA and the European Union have imposed strict regulations on their concentration in plants, both for human and animal consumption. Therefore, food plants contaminated with mycotoxins must be sorted out and destroyed. Studies by Jackson and Pryor [47] have shown that the cultivation of *P. ostreatus* on maize contaminated with aflatoxins of different concentrations (0 ng/g, 25 ng/g, 250 ng/g and 2500 ng/g) is not only possible but also leads to a degradation of the mycotoxin in the substrate of up to 94%. It is also important to note that no aflatoxins were detected in the fruit bodies of *P. ostreatus* obtained. Despite the relatively low biological efficiency of all *P. ostreatus* strains tested (up to 28.6%), no reduction in this parameter was observed even at higher concentrations of aflatoxin B1.

In some cases, it is possible to combine waste management through the cultivation of fungi with the extraction of valuable compounds. Chitin-based waste materials, such as crustacean shells, are difficult to dispose of due to their mechanical strength. The most common type of utilization is therefore collection in landfills and subsequent incineration. Xu et al. [52] have demonstrated that chitin waste shells can support *P. ostreatus* growth. Furthermore, a 1:1 combination of crayfish shells and chestnut shells can be used to obtain viable fruiting bodies of *P. ostreatus*. As no significant differences were found between the substrate variants with crayfish and the control group, this indicates the commercial potential of using mussel waste. Furthermore, the use of chitinous waste material in the growth medium resulted in almost 18% less CO_2_ emission compared to the control group.

The cultivation of *Pleurotus ostreatus* on food and agricultural waste as a substrate offers a promising strategy for sustainable mushroom production, waste utilization and environmental protection. However, the use of such waste harbors risks, mainly due to its variable and sometimes uncertain composition. Food and agricultural waste can be contaminated with pesticides, heavy metals, microplastics or pathogenic microorganisms that can accumulate in the mushrooms or remain in the spent substrate, raising concerns about biosafety during consumption and disposal [53]. The presence of inhibitory compounds in some wastes may also limit fungal growth or affect the consistency of yields. Processing and sterilization of waste substrates is critical to minimize contamination and competing microbiota. However, this process increases operational complexity and costs.

### 3.2. Synthetic Polymers

One of the few requirements placed on the fungal growth substrate is the presence of carbon, nitrogen and low levels of phosphorus and potassium. The structure of the macromolecules formed from these atoms is largely irrelevant. It is therefore possible to obtain fungi from waste materials such as synthetic polymers (Table 2). Thanks to the secretion of the enzyme laccases, *P. ostreatus* showed the ability to utilize synthetic polymers such as polyethylene or polystyrene as a carbon source [54].

Rodrigues da Luz et al. [56] investigated the possibility of cultivating *P. ostreatus* on biodegradable oxo-plastic without prior treatment with ultraviolet light or high temperature. 10 g of oxo-biodegradable polymer plastic bags were combined with 0.1 g of paper towels, hydrated, mixed with thiamine HCl and placed in a container. It was found that the oyster fungus was able to oxidize the synthetic polymer, although there was no direct substrate for the laccase, as dyes and paper were present in the substrate. However, it was surmised that the degradation of oxo-biodegradable plastic by *P. ostreatus* may combine both enzymatic and non-enzymatic reactions. More importantly, it was possible to obtain some fruiting bodies from this medium in a relatively short period of 45 days.

Non-biodegradable polymers are the main pollutants that pose a major problem in waste management. Olakanmi et al. [55] investigated the possibility of introducing disposable plastic masks into the growth substrate of oyster mushrooms. Mahogany wood shavings, wheat bran and calcium trioxocarbonate (MWS) were combined with face mask (FM) residues in different ratios: 100:0, 75:25, 50:50, 25:75 and 0:100. The shortest spawning time (25.5 days) was observed in the MWS:FM (75:25 ratio). However, mycelium growth was observed in all experimental variants, with the substrate with 100% face mask having a relatively similar spawning time (28.5 days). The highest biological efficiency (88.8%) was observed at an MWS:FM ratio of 25:75. Importantly, the fungi obtained from the growth medium with disposable face masks showed no microplastic contamination, proving that the cultivation of fungi on substrates containing plastic waste is possible.

Synthetic polymer waste is also found in used nappies in the form of polyethylene, polypropylene and superabsorbent polymers. A study by Espinosa-Valdemar et al. [57] investigated whether such nappies can be used for the cultivation of *P. ostreatus*. Nappies with and without synthetic polymers, enriched with grape pomace or not enriched, were used as substrates for the cultivation of oyster mushrooms. All variants of the experiment showed 100% colonization after 16 days. Only shredded nappies without polymers did not support the development of fruiting bodies. In terms of biological efficiency, the highest value (34.4%) was observed in the control experiment (i.e., 100% straw), but it was found that the presence of synthetic polymer’s film and the addition of grape pomace had no significant effect on this parameter.

The application potential of oyster mushroom cultivation is not limited to the production of fruiting bodies. Especially in connection with synthetic polymers, this fungus can be used as an efficient bioremediation tool. Odibgo et al. [60] tested the potential of *P. ostreatus* to degrade polyethylene terephthalate (PET) in soil and rice straw contaminated with 5% and 10% plastic fragments. Over a time of 60 days, degradation products of PET such as ketones, esters, alcohols, carboxylic acids and hydrocarbons were detected, indicating that the half-life of the plastic contained in the medium is shortened. This confirms the effectiveness of the oyster mushroom as a bioremediator for PET. Another commonly used synthetic polymer is low-density polyethylene (LDPE). The biodegradation of this synthetic polymer by *P. ostreatus* was investigated by González-Márquez et al. [61]. The liquid culture of the oyster mushroom was used to degrade different pretreated LDPE samples, including irradiated recycled LDPE (IrRPE), unirradiated recycled LDPE (UnRPE), irradiated unused LDPE (IrNPE) and unirradiated unused LDPE (UnNPE). Robust enzymatic analyses showed that the degradation of LDPE occurs by enzymatic oxidation using laccase, lignin peroxidase, manganese peroxidase and non-specific peroxygenase. Based on the activity of these enzymes and the scanning electron microscope, the highest degree of degradation was observed in irradiated recycled LDPE with a higher contact angle. Fragmentation of this type of LDPE was observed after 5 weeks. The results confirmed that irradiation and prior recycling of LDPE therefore improve the degradation process by the oyster fungus.

As mentioned above, *Pleurotus ostreatus* has shown a certain ability to degrade certain synthetic polymers. However, this biodegradation process can lead to the formation of microplastics of varying sizes, which can remain in the substrate or in the environment. This microplastic can potentially pose an ecological risk if it enters the soil or water systems during or after mushroom cultivation. Degradability by fungi varies with the size of the plastic and the composition of the substrate, with larger microplastics being more degraded due to greater contact with fungi [62]. This means that the use of waste substrates contaminated with synthetic polymers carries the risk of spreading microplastics, even if some biodegradation occurs.

### 3.3. Wastewater

Wastewater originating from the food industry is often contaminated with various pollutants and contains numerous nutrients. These properties make it a promising substitute for tap water, which is commonly used as a hydration agent for *P. ostreatus* production, as fungal growth is promoted by the enriched composition of the liquid and the load of pollutants in wastewater treatment plants can be reduced (Table 3).

Kalmış et al. [63] investigated the influence of wheat straw enriched with wheat bran soaked in tap water (control group), diluted olive mill wastewater (25%, 50% and 75%) and undiluted wastewater on the development of oyster mushrooms. The difference in spawning time between the control group and the 25% dilution of wastewater was not significant (28 days and 29.3 days, respectively). Concentrations above 25% of the wastewater led to a prolonged spawning time. The highest total yield was achieved in the control group (253 g). Increasing the concentration of the oil mill effluent by 25%, 50%, 75% and 100% led to a decrease in yield by 8.8%, 16%, 53% and 71%, respectively. Despite the reduced amount of fruiting bodies collected, the addition of 25% still allows for successful cultivation of oyster mushrooms while reducing the amount of polluted wastewater entering the wastewater treatment plants.

Atila and Kazankaya [64] investigated the influence of heavy metal-contaminated sugar factory wastewater (SMWW) on the development of *P. ostreatus*. Different concentrations of SMWW (0%, 25%, 50%, 75% and 100%) were tested as a wetting agent for the substrate of two strains (HK35 and Holl) of the oyster mushroom. The shortest spawning time for the Holl strain (19.6 days) was observed at a 50% dilution of the wastewater and for HK35 (15.4 days) without dilution (100% of wastewater). The highest yield for the Holl strain (235 g/kg) and the highest biological efficiency (67.2%) were obtained with undiluted SMWW. For the HK35 strain, the highest yield (239.2 g/kg) and biological efficiency (68.3%) were obtained with a 75% dilution of SMWW. Although there was a direct correlation between the SMWW concentration and the increase in heavy metal accumulation in the fruiting bodies, the values were still below the daily dose recommended by the WHO/FAO.

Some wastewater from food production is contaminated with pollutants that have a chemical and biological oxygen demand. One example of this is maize wastewater, the influence of which on *P. ostreatus* was investigated by Loss et al. [66]. The use of 50% maize wastewater as a wetting agent for the growth substrate of the oyster mushroom at a pH value of 5.0 resulted in a biological efficiency of 81.36%. In comparison, at a pH of 5.0 without corn wastewater, the biological efficiency of *P. ostreatus* reached 22.76%. Another method to evaluate the feasibility of wastewater application in mushroom cultivation is solid-state fermentation. Lee et al. [67] investigated the use of swine wastewater (SW) as an alternative substrate for the development of *P. ostreatus*. The wastewater was diluted to achieve the target chemical oxygen demand of 10, 15 or 20 g/L and mixed with 1.5% agar and compared with potato dextrose agar (PDA), sugar cane bagasse and wheat bran (SBWB), coffee spike extract (CHE) and whey permeate (WP). There was no significant difference in the radial growth of *P. ostreatus* between the most optimal SW variant and the PDA medium (13.5 mm/day), proving that pig waste is a viable substrate for the maintenance of oyster mushroom cultures.

The liquid culture of *P. ostreatus* can be used to remove pollutants directly from wastewater before it reaches the treatment plant. Olivieri et al. [68] investigated the possibility of removing polyphenols and total organic carbon from unsterilized olive mill wastewater using aerated flasks (400 mL capacity) and an airlift bioreactor with internal circulation (5 L capacity). The polyphenols were reduced by about 70% after 7 days without the addition of external nutrients. After their addition (in the form of glucose and yeast extract), the degradation rate increased to 95%. Analysis of the carbon balance showed that about 20% of the total organic carbon was converted into fungal biomass.

Wastewater from industrial activities contains heavy metals. Agricultural wastewater containing fertilizers, pesticides and herbicides also contribute to the heavy metal load in wastewater. It is well documented that *Pleurotus ostreatus* is able to accumulate heavy metals from its growth substrates [69]. Due to their role in mycoremediation, the fungi also take up pollutants from the substrates into their fruiting bodies. It poses a risk to biosafety when mushrooms grown for consumption are cultivated on waste substrates that contain elevated levels of heavy metals [70]. Such accumulation can lead to toxicity problems for consumers and environmental pollution if the spent substrate is disposed of improperly. The extent of accumulation also varies between metals, with cadmium and mercury often accumulating at problematic levels.

## 4. Molecular Analysis of *Pleurotus ostreatus*

Understanding the molecular biology of the oyster mushroom is crucial for assessing its commercial and scientific applications. Complete sequencing of the genome of *P. ostreatus* is now possible thanks to developments in genome sequencing technology [71,72]. This makes it possible to understand the metabolic processes, gene expression and the molecular basis of the characteristic properties of *P. ostreatus* at a deeper level. Functional genomics was used to identify important regulatory genes involved in fruiting body production, stress tolerance and environmental responses. Analyzing the transcriptome and proteome of *P. ostreatus* is gaining increasing attention. The combination of a relatively short life cycle and an abundance of biologically active compounds makes the oyster mushroom a promising candidate for molecular analysis as a model fungus.

From a commercial point of view, one of the most important parts of the *P. ostreatus* transcriptome is the genes that regulate the formation of fruiting bodies. One of the largest transcription factors—the *MYB* gene family—is responsible for numerous critical processes ranging from sporulation [73] to sexual development of the mycelium [74]. For this reason, MYB exhibits stage-specific expression patterns that change significantly at key developmental stages of the fungus [74].

Similarly to the genes from the *MYB* family, a metacaspase-encoding gene (*PoMCA1*) also influences the response to heat stress and mycelium formation. In the studies by Pei et al. [75], the expression of *PoMCA1* in *P. ostreatus* was artificially suppressed by RNA interference. The mutant strains showed slower fruiting body development, morphological deformities and increased sensitivity to heat stress compared to the wild-type strains. Conversely, overexpression of the *PoMCA1* gene in different strains resulted in increased heat tolerance and faster fruiting body development compared to the control group. Future studies need to investigate how each regulator alters transcriptional expression in order to develop a methodology for the artificial induction and regulation of critical developmental systems in *P. ostreatus.*

Breeding “sporeless” strains of *P. ostreatus* that do not produce basidiospores are crucial because of preservation of biodiversity at the genetic level. Therefore, research has focused on genes linked to meiosis, as defects in meiosis can affect basidiospore production. Lavrijssen et al. [76] recently discovered a mutation in the *MSH4* homologue of *P*. *ostreatus* that results in a null mutation that eliminates all spores. Using homologous recombination and CRISPR/Cas9, the *msh4* and *mer3* genes were targeted in order to demonstrate molecular breeding of sporeless *P. ostreatus* [77,78]. Kobukata et al. [79] identified genes involved in basidiospore production after meiotic division in *P. ostreatus*. By combining transcriptome analysis and CRISPR/Cas9 technology.

One of the most important cellular compounds of the oyster mushroom is β-glucan, which has numerous commercial applications. Understanding the molecular basis of the biosynthesis of this compound could provide a valuable framework for future genetic and methodological manipulations. Chai et al. [80] engineered a promoter for the β-1,3-glucan synthase (*GLS*) gene using the glyceraldehyde-3-phosphate dehydrogenase gene from *Aspergillus nidulans.* The mutant produced up to 131% higher yield of β-glucans than the wild type, demonstrating the influence of the *GLS* gene on β-glucan synthesis. Nesma et al. [81] analyzed the gene expression patterns in detail, focusing on the *FKS* gene, which regulates β-glucan synthesis at different developmental stages of *P. ostreatus* DMR P115 and HUC. It was found that the *FKS* gene is most frequently upregulated at the middle or end of the mycelial stage, indicating the optimal time for β-glucan extraction. The authors also showed that there are significant differences in *FKS* expression between the two strains of *P. ostreatus* studied. This gene was upregulated in both the mycelium and mature fruiting body of the DMR P115 strain. In the HUC strain, the upregulation of the FKS gene was observed in the mycelial stage.

A holistic analysis of the proteome changes can provide information on the functioning of the individual developmental stage of *P. ostreatus* fruiting body formation. Studies by Zhu et al. [82] show that the stalk has an upregulation of proteins involved in starch and sucrose metabolism, which enables more efficient transport of nutrients. The cap of the oyster mushroom showed a higher number of proteins associated with sphingolipid signaling, which in turn is related to spore production. This information will form the basis for future research to characterize specific protein activities at each stage of oyster mushroom development.

**Figure 2:**
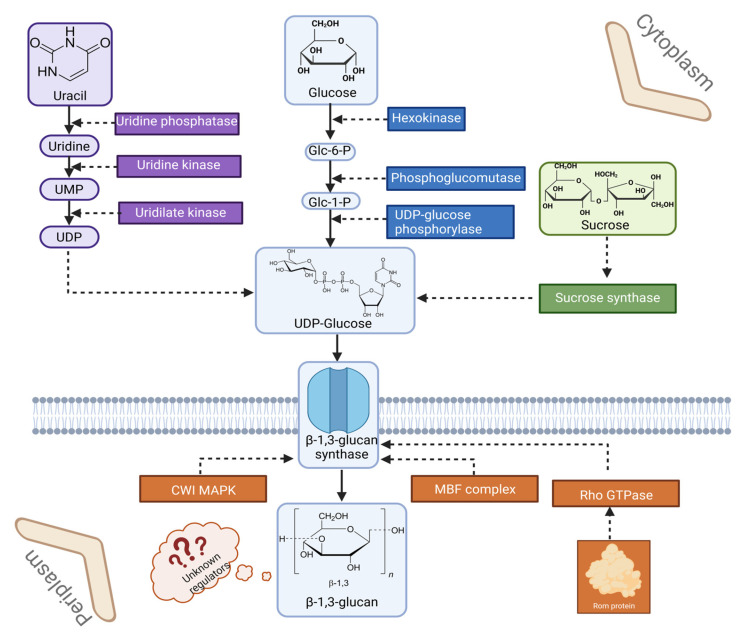
Schematic representation of β-glucan synthesis in the *P. ostreatus* cell. UMP: uridine monophosphate; UDP: Uridine diphosphate; Glc-6-P: Glucose-6-phhosphate; Glc-1-P: Glucose-1-phosphate; CWI MAPK: Cell Wall Integrity Mitogen-Activated Protein Kinase; MBF complex: Mlul Cell Cycle Box Binding Factor complex (referring to Mbp1/Swi6 complex); Rho GTPase: Ras Homologous (Rho) Guanosine Triphosphatase (prepared with Biorender.com).

Significant changes in the secretome of *P. ostreatus* can also be observed during cultivation on different substrates. Studies by Fernández-Fueyo et al. [83] have shown that oyster mushrooms cultivated on culture media supplemented with lignocellulosic materials have a drastically higher enzymatic diversity compared to purely glucose-based culture media. The amount of proteins detected and identified was also highest in strains cultivated on wheat straw (391 proteins), followed by 241 proteins in cultures on poplar wood. 206 proteins were detected on glucose medium, with carbohydrate-active enzymes accounting for the largest proportion. This result emphasizes the great diversity of metabolites produced by *P. ostreatus*. It was also proved that the proteome of the oyster mushroom can also change significantly in response to environmental conditions. One of the best-known factors that can inhibit fungal growth is CO_2_ concentration [84]. Research by Lin et al. [25] shed light on the proteomic response of *P. ostreatus* to high CO_2_ concentration. It was found that the increased CO_2_ concentration led to a shortening and thinning of the stipes, which was due to a significant down-regulation of kinases and elongation factors. In addition, a down-regulation of the protein Isp4 was observed. As this protein is related to processes of sexual differentiation, this result was inconsistent with small fruiting body stipes. This study provided information on the morphological changes in the oyster mushroom in response to a high CO_2_-enriched environment, but also highlighted the need for further analyses of proteins related to cAMP signaling and bicarbonate metabolism in this fungus. An overview of β-glucan synthesis and its regulatory context in *P. ostreatus* is shown in Figure 2.

## 5. Applicative Potential of *P. ostreatus* as a Result of High β-Glucan Contents

**Figure 3:**
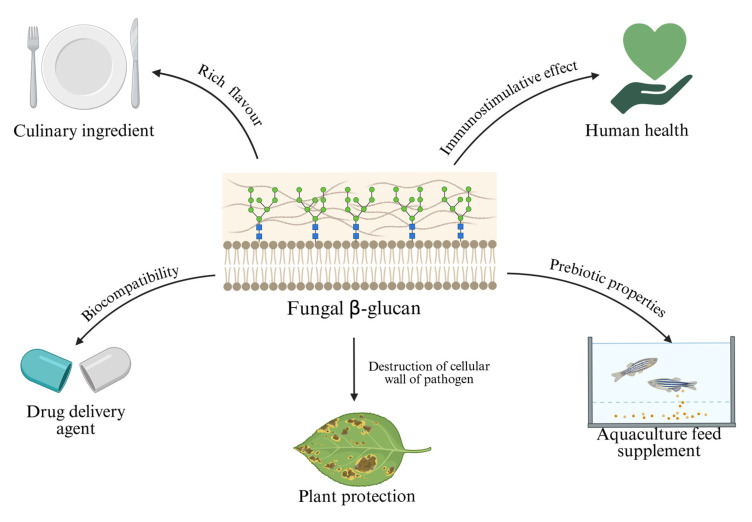
Applicative potential of β-glucan derived from *P. ostreatus*, highlighting diverse biotechnological and health-related applications including culinary use as a flavor-enhancing ingredient, immunostimulatory benefits for human health, prebiotic properties as an aquaculture feed supplement, biocompatibility enabling its use as a drug delivery agent, and plant protection by destructing pathogen cellular walls (prepared with Biorender.com).

Mushrooms have always been an important part of the human diet because they are low in calories and rich in proteins, vitamins, minerals and health-promoting bioactive compounds. Although they have long been popular in Asian countries such as Japan, Korea and China, it is only relatively recently that the rest of the world has begun to recognize the potential applications of mushrooms [85]. The popularity of the oyster mushroom stems not only from its rapid growth and high adaptability, but also from its functional ingredients, which include laccase, proteoglycan and β-glucan [8]. Of these components, β-glucan, which together with chitin forms the cell walls of the fungi, is the most widespread. The beneficial effects of *P. ostreatus* are largely attributed to this polysaccharide, mainly because this mushroom species has one of the highest concentrations of the water-soluble fraction of this substance [86]. β-Glucan has certain functionalities that can be used successfully in various industries (Figure 3).

### 5.1. β-Glucan in Human Health

Immunostimulation has been associated with β-glucan almost since its discovery [87]. However, over time it has been found that not every type of β-glucan has the same immunostimulatory properties. One of the most important parameters for the immunoreactivity of β-glucan is its solubility. Insoluble β-glucan can strongly stimulate the immune system but carries a high risk of hyperimmunity and is only suitable for oral administration. The soluble fraction of this polysaccharide also has a strong immunostimulant effect but can be administered both orally and intravenously. In addition, the risk of hyperimmunity is lower compared to its insoluble counterpart [88].

The immunostimulatory effect of soluble β-glucan in the tumor microenvironment is relatively well known. Due to its exogenous nature, β-glucan can be recognized by pathogen-associated molecular patterns with cell surface receptors on the phagocytic and cytotoxic immune cells. In response, the production of inflammatory mediators begins, which are crucial for pathogen clearance and cell maintenance [89]. Due to the heterogeneity of macrophages, they can transform into type M1 or M2. Type 1 macrophages secrete pro-inflammatory factors and have an anti-tumor effect, while M2 macrophages promote tumor growth. It has been shown that soluble β-glucan can bind to CD11b of myeloid cells and promote M1 macrophage transformation and suppress M2 transformation in the tumor microenvironment [90]. Stimulation of the immune system with soluble β-glucan can also be used to fight other pathogens. It has been shown that this polysaccharide can trigger the activation of macrophages and lead to the secretion of nitric oxide, which provides protection against *Salmonella* sp. In tests on live animals infected with the influenza virus, oral administration of soluble β-glucan showed a strong antiviral effect [91].

The immunomodulatory effect of β-glucan can also be combined with its probiotic effect. Sun et al. [92] demonstrated that vaginal administration of soluble β-glucan to mice infected with *Candida albicans* effectively controlled the disease. Soluble β-glucan of bacterial origin reduced the migration of polymorphonuclear neutrophils and thus limited inflammation. This effect is beneficial as these neutrophils cannot eliminate *C. albicans* but cause an invalid immune response. In addition, the application of soluble β-glucan resulted in the vaginal microbiome remaining almost identical in the β-glucan-treated group and the negative control group. One difference was that the group administered salecan had a higher number of enterococci, which is beneficial due to their ability to produce lactic acid, which in turn suppresses the growth of *C. albicans*.

Cardiovascular diseases are closely associated with an elevated lipid profile. A comprehensive literature review by Yu et al. [93] on the effects of oat β-glucan consumption on the lipid profile found that the consumption of soluble β-glucan significantly lowers the concentration of total cholesterol and low-density cholesterol. The lowering effect of this polysaccharide may be due to increased bile acid binding, which leads to higher cholesterol excretion and lower cholesterol absorption.

### 5.2. β-Glucan as Drug Delivery Agent

β-Glucan has an immunomodulatory effect as it is selectively recognized by pattern recognition receptors located on the surface of immune cells. The natural origin of β-D-glucan and its biocompatibility in combination with its affinity to immune cells make it a promising candidate for drug delivery [94]. *P. tuber-regium*, for example, is a source of hyperbranched β-glucan, which forms spherical molecules in aqueous solutions. These molecules can serve as stabilizers and capping agents for selenium nanoparticles thanks to their unique chemical structure that enables strong bonds between the two compounds. Combined, the average size of the constructed hybrid is 200 nm and is stable in aqueous solution for over a month. The use of soluble β-glucan as a capping agent prevents the nanoparticles from aggregating and precipitating, thus increasing their efficiency. In addition, the affinity of β-glucan to macrophages enables better targeting to tumor cells, potentially reducing the negative side effects [95].

Soluble hyperbranched β-glucan can also be used to deliver more complex compounds used in gene therapy. By combining this polysaccharide with polyethylamine (used in genetic medicine for transfection) and the desired nucleotide sequence, a biocompatible compound is created that facilitates the uptake of the desired genes into cells. In addition, the unique structure of the hyperbranched β-glucan has numerous reaction sites that enable the application of a pH-sensitive coating molecule. Additional coating reduces cytotoxicity and thus increases the possibility of accumulation at the desired sites [96]. The addition of a sensitive, releasable coating to drugs carried by soluble β-glucan will increase the accuracy of delivery to the target site while maintaining satisfactory stability of the drug. Such a delivery system would be a promising approach for prognostic biomarkers in cancer as well as for cancer immunotherapy as a whole [97].

### 5.3. β-Glucan in Aquaculture

In fish feed, β-glucan can be used as a single substance [98] or in combination with other bioactive compounds (as an adjuvant) [99,100,101,102]. There are numerous studies demonstrating the positive health effects of β-glucan in shrimp [103] and adult fish [99].

The addition of β-glucan to fish feed can improve the functionality of the digestive system, which in turn can contribute to better utilization of the feed supplied and the general condition of the farmed fish [104]. In addition, β-glucan can have an anti-inflammatory effect, which could help maintain intestinal health and counteract gastrointestinal inflammation [105,106]. The influence of β-glucan in fish includes that the intestinal epithelial cells and the length of the intestine may increase. β-glucan supplementation in the fish diet strengthens the intestinal barrier by increasing the density of goblet cells, acidomucin-secreting cells and intraepithelial leukocytes [104]. These studies suggest that β-glucan can stimulate the development of microvilli, which helps the gut to absorb nutrients more efficiently [107]. A longer intestine and a greater number of intestinal epithelial cells can contribute to better digestion and absorption of nutrients and thus improved feed efficiency. The addition of β-glucan to the diet can have a positive effect on the development of beneficial intestinal bacteria in favor of pathogenic bacteria such as *Vibrio* spp. or *Streptococcus iniae* [108,109,110,111]. Soluble β-glucan is recognized as a substance that supports the immune system of fish [112,113]. It affects the activation of immune cells such as macrophages and neutrophils, which can increase the body’s ability to fight pathogens and thus reduce the risk of diseases, especially bacterial infections. β-glucan can reduce inflammation in the body of fish, which is important for overall health, growth rate and feeding efficiency [106].

Research suggests that fish fed a diet containing soluble β-glucan have better stress resistance, which may be important under intensive aquaculture conditions [98]. The anti-inflammatory effect of β-glucan could help limit stress-induced inflammatory responses [104]. Protecting the tissue from inflammatory processes can improve the overall condition of the fish and keep it at a high level during periods of stress caused by unfavorable environmental conditions such as temperature fluctuations or an increase in nitrogenous compounds such as ammonia or nitrites [110].

## 6. Research Gaps and Challenges

Although significant progress has been made, there are still some research gaps and challenges in optimizing β-glucan production in the oyster mushroom and understanding the genetic mechanisms that control its biosynthesis.

One of the major gaps is the incomplete elucidation of the genetic and molecular mechanisms controlling β-glucan biosynthesis in *P. ostreatus*. Although individual genes such as β-glucan synthase have been identified and subjected to initial studies, the complete regulatory networks, gene interactions and environmental modulation pathways remain poorly characterized [81]. This gap limits the ability to apply precise genetic and metabolic engineering approaches aimed at increasing β-glucan yield or altering polymer structures to improve functionality. In addition, the inherent biochemical and genetic variability between *P. ostreatus* strains leads to complex β-glucan production [114]. Comparative studies on different strains are limited and there is a need for more rigorous screening and genetic characterization to identify high-yielding, robust strains suitable for industrial applications.

Cultivation parameters such as substrate composition, temperature, pH, aeration and nutrient availability significantly influence the quantity and quality of β-glucans produced by *P. ostreatus* [115]. However, systematic optimization studies using controlled bioprocessing methods are still scarce. Further research is needed to investigate different lignocellulosic substrates, including agricultural waste, and to optimize the parameters of submerged or solid-state fermentation to maximize beta-glucan yields in the long term.

The extraction and purification of β-glucans represent further research gaps. Conventional extraction methods often involve several time-consuming steps that can affect the bioactivity or yield of the polysaccharide. Although innovative methods such as microwave-assisted extraction [116] or enzymatic treatments [117] are promising, their efficacy, scalability and impact on the structural integrity of β-glucans need to be further investigated. Significant variability in the β-glucan content and quality exists between different *P. ostreatus* strains, growth conditions and developmental stages of fungal biomass [118]. This heterogeneity poses a challenge for the standardization required for pharmaceutical and nutraceutical applications. The development of reliable and rapid analytical techniques for the quantification and characterization of β-glucan remains an open area of research.

Scaling up β-glucan production from laboratory or pilot culture to industrial level is associated with hurdles related to process consistency, cost control and environmental sustainability. Efficient utilization of substrates, energy input and waste management must be balanced against maintaining product quality and yield [119]. In addition, the regulatory framework for genetically modified organisms (GMOs) poses a challenge to the implementation of advanced genetic interventions to improve varieties. Another technological obstacle is the fact that the tools for genetic modification of *P. ostreatus* are still relatively young compared to bacterial systems [12]. The lack of established, efficient transformation and genome editing systems limits the potential for targeted genetic improvements of β-glucan biosynthetic pathways. The aspect of sustainability also poses a challenge. When sourcing growing media, the environmental impact must be considered and harmonized with other agricultural uses. The development of circular bioeconomy models through the integration of agro-industrial waste streams as substrates for the cultivation of *P. ostreatus* is promising but requires further research on substrate compatibility and the impact on β-glucan quality.

Addressing these research gaps and challenges requires an integrated, multidisciplinary approach. Advances in fungi genomics, systems biology and metabolic engineering have the potential to unravel the genetic basis of β-glucan biosynthesis and facilitate strain optimization. At the same time, refining cultivation parameters through controlled experimental designs and utilizing sustainable substrates will improve the feasibility of production. Innovations in extraction technology must focus on maintaining structural integrity and bioactivity while improving efficiency. Ultimately, overcoming these challenges will support the development of scalable, cost-effective and sustainable β-glucan production from *P. ostreatus* and enable broader application in functional foods, pharmaceuticals and bioactive products.

## 7. Future Perspectives

**Figure 4:**
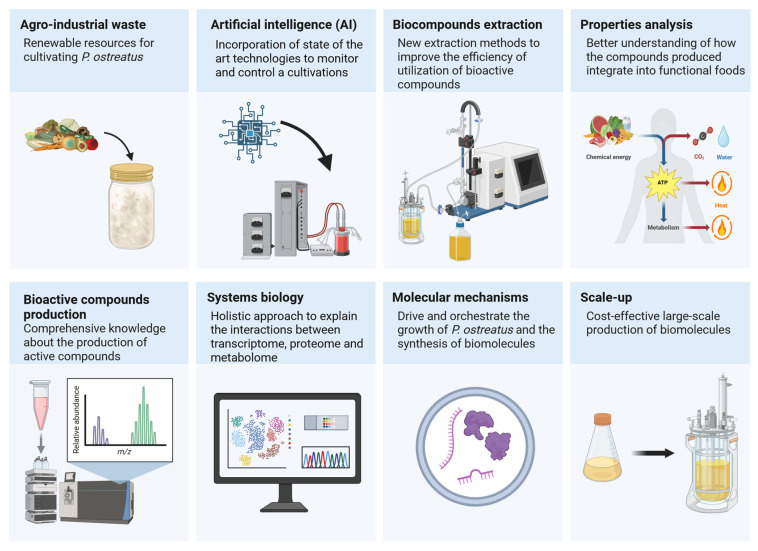
Perspectives in the cultivation of *Pleurotus ostreatus* and bioactive compounds production (prepared with Biorender.com).

The cultivation of *P. ostreatus* has made significant progress since its beginnings by Etter [120]. Similarly to the β-glucan extracted from this species, it has attracted attention for its immunostimulant effects and pharmaceutical potential. Nevertheless, there are some challenges and unexplored areas in the cultivation of oyster mushrooms that allow for further development in this area (Figure 4).

The chemical, functional and sensory properties of *P. ostreatus* are influenced by the substrates used in cultivation. Large quantities of agro-industrial waste are produced, which represent an interesting substrate due to their commercial utilization and the associated environmental problems. The idea of “converting waste into treasure” is widely accepted, leading to the repurposing of renewable resources for cultivating valuable *P. ostreatus* mushrooms. A closed-loop system in a circular economy is made possible by the growth of *P. ostreatus* on waste streams, which minimizes waste and lessens the need for virgin resources. As *P. ostreatus* appears to be able to cope with many waste materials, further comparative studies need to be carried out to achieve efficient and effective cultivation of *P. ostreatus* from renewable resources. Although the cultivation of *P. ostreatus* from renewable resources offers particular advantages, there are also significant obstacles in terms of productivity, process optimization, economics and cultivation. To overcome these obstacles, an interdisciplinary strategy involving experts from the fields of mycology, bioprocess engineering, environmental science and artificial intelligence is required. The cultivation of *P. ostreatus* using waste carbon sources is valuable from an economic and environmental perspective. The conversion of low-value waste streams into high-value *P. ostreatus* could be an important example of the industry’s transition to a circular economy.

Traditional methods have long been used for the cultivation of *P. ostreatus*, but the incorporation of state-of-the-art technologies to monitor and control the cultivation process could help to optimize this mushroom culture. Modern, emerging technologies such as the Internet of Things (IoT) and artificial intelligence (AI) techniques can advance smart food technology to increase quality and productivity in the mushroom industry. Growers could benefit from collecting and analyzing data from various sensors and cameras to remotely monitor and control environmental conditions in mushroom cultivation. AI systems are able to track environmental variables such as temperature, humidity and carbon dioxide levels to determine the ideal growing conditions for mushrooms and identify potential hazards. The AI could analyze the growth data and suggest adjustments for optimal growth of *P. ostreatus*. AI has already been successfully combined with Response Surface Methodology (RSM) to optimize parameters in the submerged culture of *P. ostreatus* [121]. Gundoshmian et al. [122] used artificial neural networks (ANN) and RSM to improve energy and water consumption to estimate the total income and environmental impact of oyster mushroom cultivation. Object detection algorithms have made rapid progress in recent years, mainly thanks to the breakthroughs achieved through deep learning [123]. Some of these algorithms go one step further and generate masks that correspond to the detected objects, a task known as instance segmentation. One of the leading tools in this category is Detectron2 [124]. In addition, YOLOv5 stands out as one of the most widely used methods for object detection, known to provide both fast and accurate results [125]. AI-assisted image analyses and machine learning algorithms have been successfully used to monitor the growth stages of *Pleurotus* spp. cultures. It was shown that the YOLOv5 algorithm can effectively identify harvestable mushrooms even in the complex environment of a greenhouse where *Pleurotus* mushrooms are cultivated. The classification and detection evaluation achieved an accuracy of up to 70% especially for fungi in their final growth stage. In addition, two models trained with Detectron2 made it possible to recognize when *Pleurotus* mushrooms had reached their maximum size and were ready for harvesting. The evaluation results showed that this method could improve harvesting decisions for up to 14.04% of the mushrooms in the greenhouse [125]. Bioreactor-based fermentation of *P. ostreatus* mycelium and metabolites benefits from ML techniques used to model and optimize operational variables. Currently, a number of bioreactor manufacturers have integrated intelligent systems to monitor and control the cultivation processes. Wang et al. [126] integrated an exhaust gas sensor into a solid-state fermentation (SSF) bioreactor and used MATLAB software (R2018a version) to analyze online data to reveal changes in CO_2_ concentration during fermentation of bread residues with *Neurospora intermedia*. Similarly, Jin et al. [127] demonstrated the use of LabVIEW software (2016 version) for real-time monitoring of CO_2_ and O_2_ concentration during SSF of crushed wheat with *Aspergillus oryzae*. The use of AI-driven soft sensors also enables real-time monitoring and automatic control adjustments based on physicochemical feedback from the culture media [128]. Gene editing technologies such as CRISPR/Cas9 have revolutionized strain improvement of *P. ostreatus*. Recent implementations include targeted gene mutagenesis through the introduction of plasmids carrying Cas9 and guide RNAs targeting the *fcy1* and *pyrG* genes. This CRISPR/Cas9 approach offers a promising tool for molecular breeding of non-genetically modified *P. ostreatus* strains and facilitates strain improvement and functional studies [129]. ML algorithms help predict the most effective guide RNA target sites and potential editing outcomes based on genomic data. In addition, AI models can analyze multi-omics datasets to identify gene targets involved in desirable traits such as rapid growth, improved β-glucan biosynthesis or stress resistance. These findings will facilitate in-built gene modifications that accelerate the development of improved *P. ostreatus* strains.

However, future developments in this area depend on the ability of researchers and producers to effectively apply advanced, innovative AI solutions. Overall, the development and utilization of AI is dependent on digital technology. AI-based intelligent mushroom cultivation enables *P. ostreatus* producers to recognize diseases or pests at an early stage and make practical suggestions for improving cultivation methods. AI-driven harvest automation makes it possible to choose the best time and technique for harvesting and thus increase production and quality.

In addition, *P. ostreatus* has attracted the attention of researchers due to its ability to produce bioactive compounds such as β-glucan. Various methods of β-glucan extraction are constantly being developed to maximize the yield of this polysaccharide. Microwave-assisted extraction and pressurized liquid extraction have been found to be more effective, shorter and use less harsh chemicals to recover β-glucan from *P. ostreatus* [116,130]. Further studies on new extraction methods are needed to improve the efficiency of utilization of this valuable compound. In addition, the data on the extraction and properties of the β-glucan obtained from *P. ostreatus* should contribute to a better understanding of how this compound can be integrated into various functional foods that offer health benefits.

The biotechnological potential of *P. ostreatus* still needs to be thoroughly evaluated in order to effectively utilize this abundant but still unknown food source on our planet. Although the technological aspects of *P. ostreatus* cultivation have been extensively studied in recent years, knowledge about its potential to produce bioactive compounds is limited. Systems biology as a holistic approach to explain the interactions between transcriptome, proteome and metabolome during the growth of *P. ostreatus* could provide comprehensive knowledge about the production of biologically active compounds. Several studies have described the genes and proteins potentially involved in *P.* sp. development and β-glucan synthesis involved [81,130,131]. However, the molecular mechanisms that drive and orchestrate the growth of *P. ostreatus* and the synthesis of biomolecules are not yet understood. Therefore, an integrated view of the different biological levels is required to understand the response of the fungus and the mechanisms leading to the biosynthesis of bioactive compounds.

## 8. Conclusions

Mushrooms have always been known not only for their flavor and nutritional value but also for their health benefits. The desirable cultivation characteristics, chemical composition and wide range of possible growth substrates of *P. ostreatus* make it one of the most important mushrooms in sustainable agriculture. The successful cultivation of the oyster mushroom requires a detailed study of its behavioral patterns, from the molecular level to the harvesting of the fruiting bodies. The data presented suggest that *P. ostreatus* can evolve from a functional food to a comprehensive mushroom-based medicine. Mushroom cultivation can be used to dispose of food waste in a sustainable way. Compared to other mushroom species, the cultivation methods of *P. ostreatus* could be simple and economical. They also help to manage environmental degradation and transform waste materials into useful bioactive molecules. This review provides important insights into the physiological needs, spawn preparation, culture substrates, additives affecting yield and nutritional and medicinal benefits of *P. ostreatus*. It also describes the available data on the genetic background responsible for the growth and development of this fungus.

## Figures and Tables

**Table 1:** Biological efficiency, yield of fruiting body, duration of spawning period of *Pleurotus ostreatus* in cultures supplemented with agricultural waste.

Substrate	Biological Efficiency (%)	Yield of Fresh Fruiting Bodies	Spawn Run Period (days)	Reference
Corncob	41.1	144.2 g/kg	28.7	[35]
Finger millet straw	50.2	253.1 g/kg	34.9	
Bamboo waste	35.1	180.8 g/kg	43.8	
100% poplar sawdust	63.6	190.9 g/kg	14.8	[36]
90% poplar sawdust + 10% green walnut husk	68.3	211.8 g/kg	14.8	
80% poplar sawdust + 20% green walnut husk	74.2	230.0 g/kg	14.8	
70% poplar sawdust + 30% green walnut husk	71.9	215.7 g/kg	14.4	
40% dry pineapple leaves waste supplement	No data	Approx. 50 g/500 g	28.0	[37]
60% dry pineapple leaves waste supplement	No data	80 g/500 g	42.0	
80% dry pineapple leaves waste supplement	No data	Approx. 30 g/500 g	48.0	
100% dry pineapple leaves waste supplement	No data	Approx. 40 g/500 g	60.0	
100% wheat straw	45.9	1.6%	No data	[38]
80% wheat straw + 10% spent coffee grounds + 10% potato peel	49.5	1.9%	No data	
70% wheat straw + 15% spent coffee grounds + 15% potato peel	57.4	1.7%	No data	
60% wheat straw + 20% spent coffee grounds + 20% potato peel	58.3	2.2%	No data	
100% soybean husk	37.2	1114.8 g	27.8	[39]
50% soybean husk + 50% sunflower husk	30.5	914.5 g	23.3	
100% sunflower seed husk	18.2	531.8 g	24.4	
33.3% soybean husk + 33.3% sunflower seed husk + 33% wheat straw	25.1	753.0 g	25.0	
50% soybean husk + 50% wheat straw	24.4	730.5 g	27.5	
50% sunflower seed husk + 50% wheat straw	17.1	514.3 g	26.0	
100% wheat straw	16.7	500.0 g	20.7	
85% corncob + 12% wheat bran + 3% lime	88.8	1420.2 g	43.3	[40]
85% corncob + 9% wheat bran + 3% beeswax waste + 3% lime	89.2	1426.9 g	46.3	
85% corncob + 7% wheat bran + 7% beeswax waste + 3% lime	92.4	1478.9 g	47.3	
85% corncob + 5% wheat bran + 5% beeswax waste + 3% lime	87.4	1398.5 g	47.0	
85% corncob + 3% wheat bran + 9% beeswax waste + 3% lime	84.2	1346.8 g	50.3	
100% coir pith	72.0	720 g	21.0	[41]
100% finger millet straw	68.0	678 g	23.0	
100% banana fiber	69.0	690 g	21.0	
100% saw dust	83.0	826 g	18.0	
100% sugarcane trash	78.0	780 g	19.0	
90% sawdust + 10% wheat bran	65.0	130.0 g	28.0	[42]
75% sawdust + 25% waste tea leaves	62.0	155.3 g	27.0	
50% sawdust + 50% waste tea leaves	79.0	189.5 g	27.0	
100% rice straw	89.0	357.4 g	29.0	
75% rice straw + 25% waste tea leaves	68.0	308.2 g	30.0	
50% rice straw + 50% waste tea leaves	64.0	258.1 g	30.0	
100% rubber sawdust	No data	140.0 g	47.0	[43]
100% paper waste	No data	70.0 g	55.0	
50% sawdust + 50% paperwaste	No data	10.0 g	55.0	
75% rubber sawdust + 25% paperwaste	No data	60.0 g	57.0	
25% rubber sawdust + 75% paperwaste	No data	23.0 g	54.0	
Hazelnut branches	63.9	255.7 g/kg	19.8	[44]
Hazelnut husks	39.4	157.5 g/kg	27.0	
Wheat straw	43.1	172.5 g/kg	20.0	
Coffee grounds	43.6	174.4 g/kg	32.7	
Rice husks	45.6	182.6 g/kg	26.7	
Hazelnut branches + Hazelnut husks (1:1)	41.6	166.2 g/kg	32.7	
Hazelnut branches + Wheat straw (1:1)	52.2	208.7 g/kg	17.3	
Hazelnut branches + Coffee grounds (1:1)	46.6	186.6 g/kg	15.7	
Hazelnut branches + Rice husks (1:1)	38.3	153.0 g/kg	17.0	
Hazelnut husks + Coffee grounds (1:1)	26.3	105.2 g/kg	45.0	
Wheat straw + Hazelnut husks (1:1)	11.3	155.3 g/kg	25.0	
Wheat straw + Coffee grounds (1:1)	59.2	236.9 g/kg	37.0	
Rice husks + Coffee grounds (1:1)	64.3	257.0 g/kg	15.0	
100% wheat straw	105.0	910.1 g/bag	32.0	[45]
67% wheat straw + 33% spent coffee grounds	105.1	814.6 g/bag	34.7	
33% wheat straw + 67% spent coffee grounds	59.3	437.1 g/bag	38.0	
80% cottonseed hulls + 18% wheat bran	54.3	4341.7 g	50.2	[46]
70% cottonseed hulls + 16% wheat bran + 12% spent mushroom substrate	61.3	4901.0 g	50.0	
60% cottonseed hulls + 13% wheat bran + 25% spent mushroom substrate	57.2	4572.0 g	51.2	
50% cottonseed hulls + 10% wheat bran + 38% spent mushroom substrate	52.9	4231.7 g	53.1	
40% cottonseed hulls + 8% wheat bran + 50% spent mushroom substrate	52.5	4198.7 g	53.9	
30% cottonseed hulls + 6% wheat bran + 62% spent mushroom substrate	48.9	3910.3 g	54.1	
20% cottonseed hulls + 3% wheat bran + 75% spent mushroom substrate	39.7	3172.0 g	54.8	
10% cottonseed hulls + 0% wheat bran + 88% spent mushroom substrate	35.9	2869.3 g	55.8	
Maize + 0 ng g^−1^ aflatoxin B_1_	21.8	No data	18.6	[47]
Maize + 25 ng g^−1^ aflatoxin B_1_	19.2	No data	23.6	
Maize + 250 ng g^−1^ aflatoxin B_1_	21.4	No data	25.8	
Maize + 2500 ng g^−1^ aflatoxin B_1_	26.7	No data	10.0	

**Table 2:** Biological efficiency, yield of fruiting body, duration of spawning period of *Pleurotus ostreatus* in cultures supplemented with synthetic polymers compared to substrates of natural origin.

Substrate	Biological Efficiency (%)	Yield of Fresh Fruiting Bodies (g)	Spawn Run Period (Days)	Reference
100% mahogany wood shavings	69.4	55.5	26.0	[55]
75% mahogany wood shavings + 25% face masks	76.9	55.6	25.5	
50% mahogany wood shavings + 50% face masks	6.3	5.0	28.0	
25% mahogany wood shavings + 75% face masks	88.8	71.0	29.0	
100% face masks	68.8	55.0	28.5	
Diaper cores and coffee waste in ratio of 5:1	No data	84.0	26.0	[49]
Diaper cores and coffee waste in ratio of 6:1	No data	55.0	28.0	
Diaper cores and coffee waste in ratio of 7:1	No data	49.0	31.0	
Plastic bags (oxo-biodegradable)	No data	No data	45.0	[56]
Diaper with plastic, ground + grape	12.6	No data	16.0	[57]
Diaper without plastic, ground + grape	19.3	No data	16.0	
Sawdust + 150 mL of crude oil	No data	272.5	14.0	[58]
Sawdust + 100 mL of crude oil	No data	312.3	14.0	
Sawdust + 50 mL of crude oil	No data	320.6	14.0	
Corncob spawn	70.9	186.2	34.6	[59]
Sugarcane bagasse spawn	69.2	181.6	35.4	
Loofah sponge spawn	70.1	184.0	35.6	
Polyurethane spawn	68.7	180.2	36.1	
Sawdust spawn	70.2	184.3	34.4	

**Table 3:** Biological efficiency, yield of fruiting body, duration of spawning period of *Pleurotus ostreatus* in cultures supplemented with wastewater.

Substrate	Biological Efficiency (%)	Yield of Fresh Fruiting Bodies	Spawn Run Period (Days)	Reference
100% tap water	50.7	253.0 g	28.0	[63]
25% olive mill effluent + 75% tap water	46.1	231.0 g	29.3	
50% olive mill effluent + 50% tap water	42.6	212.0 g	35.8	
75% olive mill effluent + 25% tap water	23.8	119.0 g	37.8	
100% olive mill effluent	14.7	73.2 g	43.8	
0% sugar mill wastewater	54.6	196.5 g/kg	26.0	[64]
25% sugar mill wastewater	64.0	224.0 g/kg	26.6	
50% sugar mill wastewater	63.6	222.8 g/kg	27.2	
75% sugar mill wastewater	68.3	239.2 g/kg	29.9	
100% sugar mill wastewater	67.5	236.3 g/kg	27.6	
Coffee residue + wheat straw + 20% lipid fermentation wastewater	64.5	168.9 g	22.0	[65]
Coffee residue + beech wood shavings + 20% lipid fermentation wastewater	64.6	151.8 g	22.0	
Olive crop + wheat straw + 20% lipid fermentation wastewater	72.2	176.2 g	22.0	
Olive crop + beech wood shavings + 20% lipid fermentation wastewater	71.5	174.4 g	20.0	
Rice husk + wheat straw + 20% lipid fermentation wastewater	62.4	73.6 g	24.0	
Rice husk + beech wood shavings + 20% lipid fermentation wastewater	63.2	79.0 g	25.0	
Wheat straw + tap water	79.7	279.1 g/bag	No data	[34]
Wheat straw + coffee residue + tap water	76.9	269.3 g/bag	No data	
Wheat straw + coffee residue + olive crop + tap water	77.6	271.6 g/bag	No data	
Wheat straw + olive crop + tap water	72.0	252.1 g/bag	No data	
Wheat straw + lipid fermentation wastewater	76.6	268.1 g/bag	No data	
Wheat straw + coffee residue + lipid fermentation wastewater	72.9	255.3 g/bag	No data	
Wheat straw + coffee residue + olive crop + lipid fermentation wastewater	82.2	287.6 g/bag	No data	
Wheat straw + olive crop + lipid fermentation wastewater	77.4	270.9 g/bag	No data	
